# Refractory Cytomegalovirus Colitis in Common Variable Immunodeficiency Requiring Total Colectomy

**DOI:** 10.1155/2023/8888429

**Published:** 2023-12-21

**Authors:** Sulaiman Almushir, Faisal Aljohani, Abdulrahman Qatomah

**Affiliations:** ^1^Division of Gastroenterology and Hepatology, McGill University Health Centre, Montreal, QC, Canada; ^2^Department of Internal Medicine, Tabuk University, Tabuk, Saudi Arabia; ^3^Division of Gastroenterology and Hepatology, King Faisal Specialist Hospital and Research Center, Jeddah, Saudi Arabia

## Abstract

Cytomegalovirus (CMV) colitis is an uncommon infection in immunocompetent hosts, usually occurring in the presence of an underlying immunodeficiency condition that allows for the reactivation of latent CMV infection. CMV colitis typically presents with persistent diarrhea, sometimes accompanied by bloody stools and nonspecific abdominal pain. We present the case of a 76-year-old woman known to have chronic CMV colitis, which was diagnosed in the context of underlying common variable immunodeficiency (CVID). Despite multiple attempts at managing CMV colitis, her symptoms persisted over the years. Ultimately, the patient required a pan colectomy due to refractory CMV colitis.

## 1. Introduction

Cytomegalovirus (CMV) is a double-stranded DNA virus that belongs to the herpesvirus family. It is a virulent virus that can affect multiple organs, including the colon, retina, esophagus, and the central nervous system (CNS) [1]. However, most of these infections occur in the context of underlying immunodeficiency conditions, such as acquired immunodeficiency syndrome (AIDS), organ transplant patients, patients undergoing chemotherapy, and individuals with underlying immunodeficiency diseases like common variable immunodeficiency (CVID) [2, 3]. Healthy individuals can also contract CMV infections; however, the complications are more severe in immunodeficient hosts when compared to healthier individuals.

CMV colitis is an infection of the colon that typically presents with nonspecific symptoms such as diarrhea, unexplained weight loss, and fever. Diagnosing CMV colitis can be challenging due to its clinical presentation, which is similar to other common gastrointestinal conditions. Furthermore, CMV colitis may exhibit endoscopic features resembling other inflammatory diseases, such as inflammatory bowel disease (IBD), leading to potential misdiagnosis. However, when considering the entire clinical picture, particularly in cases of known immunocompromised patients, a high suspicion of CMV colitis, after excluding other common conditions, is a reasonable differential diagnosis. Accurate diagnosis is crucial, given the distinct management approaches for CMV colitis compared to similar conditions like IBD [4, 5].

## 2. Case Report

**Figure 1:**
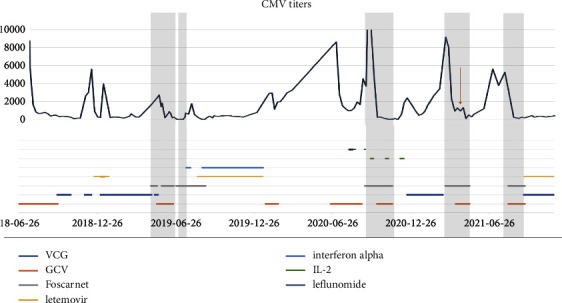
CMV titer and corresponding antiviral medication received throughout the course of the disease. VCG: valganciclovir. GCV: ganciclovir. IL-2: interleukin-2 (aldesleukin). CMV titer: copies/ml.

**Figure 2:**
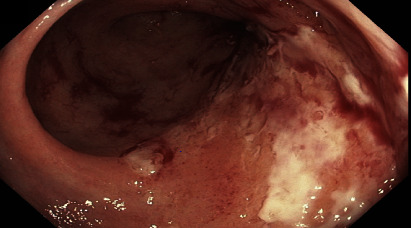
Endoscopic image showing colonic ulceration.

**Figure 3:**
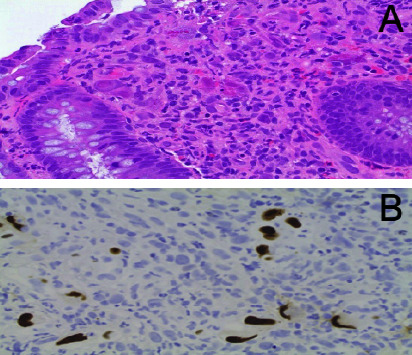
Inclusion bodies on colonic biopsy with positive immunohistochemistry for CMV.

A 76-year-old woman, known to have cytomegalovirus (CMV) colitis diagnosed through a colonoscopy with colonic biopsy that tested positive for CMV, presented with ongoing diarrhea and rising CMV titers despite medical treatment with ganciclovir, foscarnet, and weekly IgG infusions. Over the course of four years, the patient had multiple hospital admissions. Ganciclovir resistance testing revealed a mutation, D843D/V, in the UL54 region, possibly causing resistance to ganciclovir, foscarnet, and cidofovir. Subsequently, she underwent various first-line, second-line, and investigational therapies, including interferon alpha, intravenous foscarnet, intravenous ganciclovir, valganciclovir, letermovir, leflunomide, and multiple cycles of interleukin-2 (aldesleukin) injections. Despite receiving multiple lines of treatment, her symptoms persisted, with worsening diarrhea and abdominal pain that required multiple emergency room visits. Her CMV titers remained elevated (Figure 1). Repeat CT scans of the abdomen and pelvis showed progression and worsening colon thickening, involving the entire colon and cecum compared to earlier scans that indicated involvement of only the ascending colon and cecum. A repeat colonoscopy (Figure 2) revealed pan colitis with ulceration, and the biopsy confirmed severe colitis with positive CMV inclusion bodies and immunohistochemistry from multiple sites of ulceration and inflammation (Figures 3(a) and 3(b)). The patient underwent a trial of fresh frozen plasma, which was unsuccessful. After exhausting all medical treatment options and due to the worsening of CMV colitis, a multidisciplinary decision was made to proceed with a pan colectomy, which was performed around mid-2021, as indicated by an arrow on Figure 1. Initial postoperative CMV titers remained elevated (Figure 1), but subsequent measurements ranged between 1000 and 2000 copies/ml, resulting in significant clinical improvement.

## 3. Discussion

In our case, the patient had been diagnosed with common variable immunodeficiency (CVID) at the age of 60 and had been receiving weekly IgG injections without antibiotic prophylaxis. The diagnosis of CVID was made in the context of recurrent pneumonia episodes occurring 3-4 times per year. Given the patient's known CVID and ongoing care under an immunologist, the medical team suspected an underlying CMV colitis. During the initial colonoscopy, there was still suspicion of a concomitant inflammatory condition, such as ulcerative colitis or Crohn's disease, as the initial biopsies suggested idiopathic inflammatory bowel disease with findings like cryptitis and architectural distortion. However, subsequent biopsies, along with immunohistochemistry, confirmed CMV colitis, and the possibility of underlying IBD could not be ruled out. The pathologist suggested repeating the biopsy once the CMV infection was treated. After consulting with infectious disease experts, the decision was made to actively treat the patient. Multiple lines of management, as mentioned earlier, were attempted. Unfortunately, poor clinical response, increasing CMV titers, disease progression on imaging, and endoscopic evidence of active inflammation led the medical team to approach this case in a multidisciplinary manner. The decision was made to proceed with a pan colectomy and end ileostomy, which resulted in a favorable outcome. This decision was based on several factors. First, the patient had an exceptionally resistant form of CMV colitis that did not respond to any medical management, as evidenced clinically, biochemically, on imaging, and endoscopically. Second, the benefits of surgical intervention outweighed the risks, primarily the risk of perforation and eventual death [6]. Furthermore, no other medical management options were available, as all potential treatments had been exhausted without success. Based on this case report, we would recommend considering pan colectomy with end ileostomy as a viable option when all medical management options have been exhausted and there is no improvement in the patient's condition.

## Data Availability

All data generated or analyzed during this study are included in this article. Further enquiries can be directed to the corresponding author.
